# Metabolomic Fingerprinting for the Detection of Early-Stage Lung Cancer: From the Genome to the Metabolome

**DOI:** 10.3390/ijms23031215

**Published:** 2022-01-21

**Authors:** Jean-François Haince, Philippe Joubert, Horacio Bach, Rashid Ahmed Bux, Paramjit S. Tappia, Bram Ramjiawan

**Affiliations:** 1 BioMark Diagnostics Inc., Richmond, BC V6X 2W8, Canada; jhaince@biomarkdiagnostics.com (J.-F.H.); Rashid.biomark@gmail.com (R.A.B.); 2 Institut Universitaire de Cardiologie et de Pneumologie de Québec, Department of Pathology, Laval University, Quebec, QC G1V 4G5, Canada; philippe.joubert@criucpq.ulaval.ca; 3 Department of Medicine, Division of Infectious Diseases, University of British Columbia, Vancouver, BC V6H 3Z6, Canada; horacio.bach@gmail.com; 4 Asper Clinical Research Institute, St. Boniface Hospital, Winnipeg, MB R2H 2A6, Canada; bramjiawan@sbrc.ca; 5 Department of Pharmacology & Therapeutics, Rady Faculty of Health Sciences, University of Manitoba, Winnipeg, MB R3E 0T6, Canada

**Keywords:** lung cancer, diagnosis, molecular targets, proteomics, metabolomics, early detection

## Abstract

The five-year survival rate of lung cancer patients is very low, mainly because most newly diagnosed patients present with locally advanced or metastatic disease. Therefore, early diagnosis is key to the successful treatment and management of lung cancer. Unfortunately, early detection methods of lung cancer are not ideal. In this brief review, we described early detection methods such as chest X-rays followed by bronchoscopy, sputum analysis followed by cytological analysis, and low-dose computed tomography (LDCT). In addition, we discussed the potential of metabolomic fingerprinting, compared to that of other biomarkers, including molecular targets, as a low-cost, high-throughput blood-based test that is both feasible and affordable for early-stage lung cancer screening of at-risk populations. Accordingly, we proposed a paradigm shift to metabolomics as an alternative to molecular and proteomic-based markers in lung cancer screening, which will enable blood-based routine testing and be accessible to those patients at the highest risk for lung cancer.

## 1. Introduction

Cancer is a leading cause of death worldwide, accounting for nearly 10 million deaths in 2020 [1]. Among the most common global cancer-related death in 2020, lung cancer accounted for 1.8 million deaths. In addition, there were 2.21 million new cases of lung cancer around the world [1]. According to the World Cancer Research Fund, lung cancer is the most common cancer in men and the third most common cancer in women [2]. Although the American Cancer Society recommends that those over 40 years undergo yearly cancer check-ups, this recommendation is usually not followed. Consequently, survival tends to be poorer, when patients show cancer symptoms at a later stage of development [3].

The early diagnosis of lung cancer is key to a successful treatment regimen and improved prognosis; however, current early detection methods for lung cancer are not adequate. Furthermore, some of these technologies used in those methods are invasive, cause discomfort and pain to the patients and may present a greater risk of complications. Personalized disease risk based on genomic information has assisted therapeutic decisions. However, genomics remains relatively limited in predicting disease onset, mainly because genomic information does not account for the dynamic environmental influences (phenotype). Thus, to better understand lung cancer, the examination of downstream changes occurring at the level of the protein and metabolites might provide helpful information about the disease. Although there are a number of recent reviews on the topic of metabolomics in human health and disease [4,5,6,7,8,9,10,11,12,13], there is a paucity of information regarding the utility of metabolomics as a robust cancer diagnostic platform. Accordingly, the purpose of this brief review was to chart a general cancer diagnostic landscape and various approaches in the diagnosis of lung cancer, but with a major focus on the use of liquid biopsy. We also highlighted the role of metabolomics and how it has been underutilized as a tool in cancer diagnostics, particularly for the detection of early-stage lung cancer. We, therefore, searched PubMed for primary research articles regarding the potential of clinical applications of metabolomics. Specifically, the search strategy used the following search terms for relevant articles over the last 10 years: biomarkers, metabolomics, lung cancer, early-stage lung cancer, and liquid Biopsy.

## 2. Challenges and Importance of Better Screening Approaches for Early-Stage Lung Cancer

The two main types of lung cancer are small-cell lung carcinoma (SCLC) and non-small-cell lung carcinoma (NSCLC), with NSCLC accounting for 85–90% of observed cancers [14,15]. The vast majority (85–90%) of lung cancer cases are due to long-term tobacco use, while 10–15% of cases occur in people who have never smoked [16]. Non-smokers typically develop lung cancer through exposure to radon gas, asbestos, second-hand smoke, air pollution, toxic metals, soot, sawdust, and/or coal dust [17,18,19,20]. The typical age at diagnosis is 70, with slightly more men diagnosed than women [21]. The five-year survival rate of lung cancer patients is approximately 21%, mainly because most newly diagnosed patients present with advanced or metastatic disease.

Lung cancer is histologically categorized into adenocarcinoma, large-cell carcinoma, and squamous cell carcinoma. The stages of lung cancer are based on the staging system established by the American Joint Committee of Cancer (AJCC). The staging system is acronymized as TNM, where T stands for the size of the primary tumor, N stands for the spread of the tumor to lymph nodes, and M stands for metastasis. Despite decades of research and the introduction of many advanced therapeutics, survival rates for lung cancer have remained essentially unchanged [22]. However, when lung cancer is detected at stage I (T < 5 cm, no spread to lymph nodes, no metastasis), the 10-year survival rate increases to 88%, and if the tumor is resected within one month of detection, the survival rate is 92% [23]. The five-year survival rates for NSCLC have been reported to range from 14% to 49% for stage I to stage IIIa lung cancer and <5% for stages IIIb/IV (Table 1), but with the advancement of treatment regimens, some improvements have been observed [24].

Although advances in multimodality therapeutics for lung cancer have been made, the overall five-year survival rate among newly diagnosed lung cancer patients at a late stage remains in the range of 15–17% [26,27]. Although surgical resection is the treatment of choice for early-stage NSCLC, if lung cancer is detected at an early stage, the 5-year survival is reported to range from 36% to 70% [28,29,30]. On the other hand, multimodality treatment with adjuvant chemotherapy has improved survival rates by only 5% [28,30]. It should be noted that until the last decade, the five-year overall survival rate for patients with metastatic NSCLC was <5%; however, with an improved understanding of the pathophysiology of lung cancer, the overall survival rate has improved to 25% to 40% [31]. Since 90% of lung cancer cases are detected among smokers and former smokers, this high-risk population group would benefit from a screening test with the goal of detecting lung cancer while it is in stage I [32] or earlier.

These data strongly support the contention that early diagnosis is fundamental to the successful treatment of lung cancer. Unfortunately, current early detection methods of lung cancer are not ideal. These methods include chest X-rays, bronchoscopy, sputum analysis followed by cytological analysis, and low-dose computed tomography (LDCT). Although radiation exposure during chest X-rays may be problematic, a major reason for not using X-rays may be attributed to the poor performance of the test concerning sensitivity and specificity. Accordingly, as of 2016, the Canadian Task Force on Preventative Health Care (CTFPHC) has recommended against using chest X-rays for lung cancer screening [33]. Some of the issues with bronchoscopies are that they are invasive, not readily available and associated with potential complications. In addition, false-negative bronchoscopic results are commonly experienced, and the diagnostic accuracy of this technique is sub-optimal, with sensitivities ranging between 34% and 88%, particularly for the diagnosis of peripheral malignant lesions [34]. Indeed, these sensitivities depend on the size and the localization of the primary tumor and the number of parallel tests performed per bronchoscopy [35,36,37].

The diagnosis of lung cancer using standard cytological sputum analysis can be tedious, prone to unsatisfactory sample collection and often exhibits poor sensitivity [38]. To achieve a diagnosis, indeterminate sputum results may necessitate repeated sputum collection or escalation to more costly and invasive testing methods. LDCT, while more sensitive than chest X-rays, is expensive, exposes patients to a higher radiation dose than chest X-rays, is not routinely accessible to many patients and has a 96% rate of false positives (with a 4 mm non-calcified nodule size cut-off) [39]. In an assessment conducted by the National Lung Screening Trial in the USA, the over-diagnosis rate of lung cancer by LDCT was estimated to be 18.5% [40]. LDCT screening can thus lead to invasive interventions (needle biopsy and/or surgery) [41] for many patients with benign lesions, leading to patient morbidity and high health care costs. Additionally, cumulative radiation exposure from repeated scans may increase the risk of developing cancers [42]. Indeed, a widespread issue with LDCT lung cancer screening is the potential harm attributed to exposure to ionizing radiation as well as in cases during the work-up of suspicious lesions discovered at LDCT [43]. However, in the Continuous Observation of Smoking Subject (COSMOS) lung cancer study, it was found that one radiation-induced major cancer would be expected for every 108 (259/2.4) lung cancers detected through screening. It was concluded that radiation exposure and cancer risk associated with lung cancer LDCT screening are not insignificant but acceptable due to a highly significant reduction in mortality achieved with screening [44]. Nevertheless, a 20% reduction in lung cancer mortality has been reported using intensive LDCT screening of heavy smokers [45].

It should be mentioned that the Nederland’s Leuvens Longkanker Screenings Onderzoek (NELSON) trial was conducted based on a volumetry-based screening strategy [46]. Of the lung cancers diagnosed, around 71% were diagnosed at stage I and approximately 8% at stages IIIb–IV, and 51.2% were adenocarcinomas, thus demonstrating that, unlike other comparable trials, the NELSON trial screen-detected lung cancers are more often diagnosed at stage I and less frequently at stages IIIb–IV. However, it was suggested that the screening strategy of the NELSON trial results in a favorable diagnosis [46] with a reduction in mortality from lung cancer [47]. Still, the complete consensus for a systematic lung cancer screening is yet to be attained [47].

## 3. Molecular Approaches and Directions in Lung Cancer Detection

Given the limitations detailed above, it is clear that better, less expensive, less invasive, and broader screening approaches to early lung cancer detection are urgently needed. To address this need, many researchers have looked to the development of non-invasive or mildly invasive molecular tests using breath [48], urine [49], sputum [50], breath condensate [51], or blood-based assays [52]. The molecular markers being used include proteins [53,54], tumor-associated autoantibodies [55], circulating DNA [56], circulating tumor cells [57], microRNA [58], methylated DNA [59], and metabolites [60]. Many of these reports tend to focus on diagnosing late-stage lung cancer as opposed to early-stage lung cancer. Among those focused on early-stage lung cancer, some of the better molecular tests reported the area under the receiver operating characteristic (AUROCs) curves of 0.84 [61,62] to 0.90 [63]. To date, very few molecular tests have been validated on larger populations. Only one molecular blood test for early-stage lung cancer has reached the market; unfortunately, this test only shows a 37% sensitivity and a 90% specificity [64,65]. However, it should be noted that a randomized ongoing clinical trial led by Oncimmune and Lung Cancer Scotland (ECLS) was designed to confirm these findings [66]. Dama et al. [67] have recently reviewed some of the current technologies used in the early detection of lung cancer. Overall, liquid biopsy has shown some clinical applicability as a tool for the early detection of lung cancer, and its use as a screening/diagnostic/prognostic test for cancer detection remains to be fully validated for implementation in clinical practice [68].

## 4. The Interrelationship between Genes, Proteins, and Metabolites

There are a number of factors that can influence the metabolome of an individual, including age, sex, diet, geographical location, environment, ethnicity, time of day, and even the individual’s endogenous genetic make-up [69,70]. Metabolites and genes are intimately connected [71]. In this regard, it has been suggested that a single DNA base change in a given gene can lead to 10,000-fold endogenous metabolite shift levels [72]. This substantial increase in metabolite concentration is attributed to the fact that metabolites are the downstream products and interactions of multiple intracellular elements, including genes, transcriptional activators, RNA transcripts, protein transporters, and enzymes [73].

**Figure 1:**
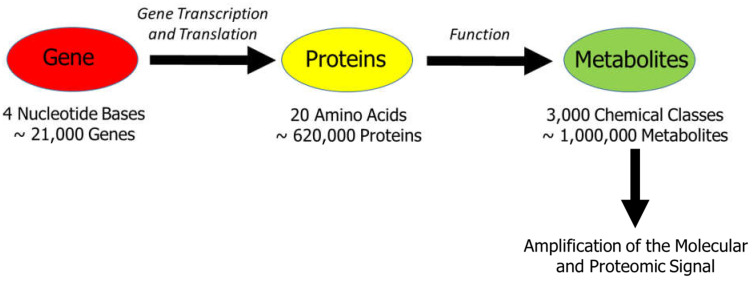
The interrelationship between genes, proteins, and metabolites. This scheme illustrates the amplification of the metabolomic signal, which involves the initial message transfer from DNA to functional proteins and then organ-specific metabolites. Adapted from Wishart, DS [72].

This amplification of the metabolomic signal, depicted in Figure 1 (adapted from [72]), involves the transmission of the message from DNA to proteins to metabolites. In other words, it can also be described in terms of the number of genes (20,000) versus the number of (expected) metabolites (~1 million) and the diversity of chemicals used to assemble genes (4 nucleotides) or proteins (20 amino acids) versus the variety of chemicals seen in the metabolome (3000 chemical classes) [72]. The organs of the body produce or utilize specific metabolites that can serve as metabolomic fingerprints in health and different pathophysiological conditions [72]. Thus, metabolomic fingerprinting provides a unique opportunity for the use of metabolites as a panel of biomarkers of different diseases, including cancer, which can exhibit highly specific metabolic signatures that have both diagnostic and prognostic value.

## 5. Metabolic Fingerprints

It has been known for a long time that cancer can be regarded as a metabolic disease [74]. Since metabolism reflects the biochemical state of a healthy or unhealthy cell, then the unique metabolic characteristics (fingerprints) may assist in the determination of the stage and location of a carcinoma in a non-invasive manner and allow for the distinction between metabolic processes in healthy cells as well as cancer cells [74]. Such metabolic disorder helps to support high proliferative rates despite poor vascularization that limits oxygen and nutrient supply [75,76]. Accordingly, the field of cancer metabolomics is primarily based on the fact that metabolic activities are altered in cancer cells relative to normal cells that represent a characteristic metabolic profile that sustains malignancy and is thus considered a hallmark of cancer [77,78]. In cancer cells, biochemical pathways are disturbed due to the disruption of gene regulation and genetic polymorphisms, unusual chromosome repetitions, and mutations [78,79]. These disruptions may occur within the pathway itself or from downstream effects caused by dysfunctional proteins in adjacent pathways. Dysfunctional proteins in pathways are often related to cancers and can cause an imbalance in metabolite concentrations that are different from healthy cells [72,74]. It is thus conceivable that each other type of cancer may be caused by different mutations (differing dysfunctional proteins) with unique metabolomic profiles. However, the exact nature and mechanisms responsible for metabolic reprogramming in cancer cells remain to be fully explored [79].

From the aforementioned, lung cancer diagnosis generally occurs at the late stages of the disease, when curative treatments are not efficacious or possible anymore. Thus, new methodologies were explored to identify early signs of the disease. One of them, the presence of specific metabolites in different body fluids, might offer an answer for an early diagnosis. Metabolomics has been used to detect and quantify metabolites related to cancer. Fluids such as serum, plasma, urine, and sputum have been evaluated as potential sources of specific metabolites. A summary of the studies that evaluated the metabolomics of lung cancer versus healthy controls using body fluids is described in Table 2. In these studies, metabolites with sufficient power to discriminate the lung cancer groups from the healthy controls were indicated after statistical analyses. Although this finding constitutes a significant advance in discovering universal biomarkers, no matching was observed when comparing the metabolites between the studies.

Furthermore, although changes in similar biochemical pathways were observed, no specific metabolite(s) have been found, as represented in all of the studies. It should be mentioned that while the use of various fluids such as serum, plasma, urine, and sputum for metabolic studies has been described, another important fluid proposed more recently for lung cancer studies is bronchoalveolar lavage fluid (BALF), due to its vicinity to cancerous tissue. In this regard, technological advances in separating and detecting exfoliated tumor cells from BALF for lung cancer diagnosis have recently been reported [97]. In addition, cell-free DNA (cfDNA) from BALF has also been used to identify lung cancer. Indeed, cfDNA profiling can distinguish small malignant tumors (≤2cm in diameter) from benign pulmonary nodules with a reported 83% sensitivity and an 87.5% specificity, thus potentially being of diagnostic value [98]. In addition, exhaled breath condensate (EBC) and breath biomarkers for lung cancer have also been proposed as a testing sample for lung cancer diagnosis [99]. However, the cost and reproducibility are concerning. Furthermore, interference effects and staging of patients for early detection still need to be explored to establish the clinical applicability of metabolomics of EBC and breath biomarkers in lung cancer diagnostics.

It should be noted that the data presented in Table 2 reflect the dysregulated metabolites in different biofluids, even though the metabolomics of lung cancer tissue were also reported in some of these studies. It is the intent of this review to focus on biofluids only as the trend is now on liquid biopsy as a valuable diagnostic tool. However, finding universal biomarkers for disease diagnosis is difficult because of the complexity of the analyzed fluids, especially serum and plasma. In addition, the lack of a standardization protocol generates variations between the studies. Some of the concerns in the performance of the studies are as follows.

### 5.1. Variations of Metabolites

Metabolite variation in individuals has been analyzed, and the need for thousands of patients has been suggested to obtain robust epidemiological studies [100]. Metabolite levels could fluctuate daily during the year and significantly if diets are changed. Generally, studies do not assess the effect of ethnic groups of the cohort, and the variability in the studies can be caused by different nutrients. In addition, biochemical pathways may undergo changes based on ethnicity [101,102].

### 5.2. Collection, Storage, and Processing

The stability of metabolites during collection until the analysis is critical to obtain consistent results. For example, plasma was shown to be more stable than serum, and other factors should be considered, such as clotting time, ambient temperature, and freezing-thawing cycles [103]. Moreover, studies have shown that glycolysis intermediates, amino acids (e.g., histidine), acetate, and diacylglycerol levels might be compromised by this processing [104]. In the case of urine samples, different profiles were observed, when the same sample was stored at 4 °C and −20 °C. The results showed a significant reduction in 14 metabolites (e.g., *N*-acetyl glycine, adenosine, creatine, pyridoxal, and succinic acid) in samples stored at 4 °C compared to a more stable level at −20 °C [105].

### 5.3. Variations of Manufacturing Kits

The comparison of the results obtained by the platforms Biocrates and Metabolon showed some discrepancies that should be considered a metabolite variation. For example, the metabolite lysoPC a C20:4 showed a low correlation with the same cohort results [106]. This variation probably originated from the way both platforms analyzed this specific metabolite. For example, Metabolon quantified lysoPC 20:4 fatty acid chain at position *sn1*, whereas Biocrates did not discriminate between positions *sn1* and *sn2* and it quantified only the total levels of both fatty acids.

### 5.4. Instrumentation and Data Processing

Two types of mass spectrometry (MS) instruments are used in metabolomics. They include gas- and liquid-chromatography (GC and LC, respectively). Upon measuring the masses of the metabolites, a library is necessary to determine the identity of each one. This analysis represents a potential impact on data analysis and processing, as different libraries are available. Each one has different algorithms that might bring slight differences in a study. For example, the libraries accurately identify known metabolites but may be less efficient for unknown molecules.

Other factors in the performance of the instruments are related to the analysis of hundreds of samples that might bring differences based on the instrument performance. For example, analytical changes result from column degradation, GC/LC conditions, mass spectrometer contamination, or metabolite degradation/decay due to the waiting time [107,108].

### 5.5. Effect of Underlying Diseases or Non-Related Metabolites

It is important to determine the impact of medications on underlying diseases/conditions when analyzing metabolomics results. For example, therapies related to underlying diseases in lung cancer comorbidities are metabolized mainly in the liver. This metabolism could generate novel metabolites by introducing errors in the metabolomic analysis. In addition, the microbiome might also metabolize drugs that can be reabsorbed in the bloodstream [109], introducing confounding factors. Other examples include the presence of paracetamol as a metabolite with high significance, which is probably the residual of a drug consumed by the patients [87].

Moreover, the presence of bisphenol A in the list of metabolites is problematic as this is a synthetic molecule, and its presence can be related either to an error in the identification of the molecule or, less probably, to high-consumption bottle beverages as bisphenol A is used in the manufacturing of polycarbonate bottles. In addition, as bisphenol A is poorly soluble in water, the presence in blood can result from high levels in drinking water [92]. Finally, it should be pointed out that a number of different factors need to be considered when undertaking metabolomic studies, including diet [110] and comorbidities [111,112,113,114,115,116,117,118,119]. In this regard, obesity [111,112], chronic obstructive pulmonary disease [113,114,115], kidney disease [116], diabetes [117,118], and cardiovascular disease [94] have all been reported to have a major impact on the metabolome that can confound metabolomic studies in cancer, and thus, their influence cannot be underestimated.

Although the application of metabolomics is growing for cancer diagnosis, more studies are necessary to be implemented for a final use for biomarker measurement. As mentioned above, there is an inconsistency in the chosen metabolites that discriminate lung cancer from healthy individuals. In addition, the heterogeneity of samples related to the types of lung cancer, together with the different stages in the patients, is adding more variability. Overall, the validation of new biomarkers in lung cancer diagnosis needs studies that include thousands of patients stratified according to their cancer types and stages of the disease and effects of other clinical parameters, including comorbidities and drug interactions.

We have focused on the polyamine pathway due to its connection to many types of cancer. Multiple proteins constitute this pathway, and when one of them does not function properly, the levels of metabolite at the point of dysfunction will be altered until they reach a new equilibrium that allows cancer to grow. Using this concept, we have defined and validated the preliminary fingerprint for lung cancer and are in the process of determining the fingerprint for breast cancer. Using quantitative metabolite tests instead of genetic tests, protein tests, or X-ray imaging allows such a test to be quickly and inexpensively integrated into the existing clinical testing infrastructure. It also makes the test far more reproducible and much more accurate [96]. As of yet, no other high-performing chemical/metabolite test for lung cancer screening has been developed anywhere in the world.

## 6. Potential Impact on Patients

The Canadian Task Force on Preventative Health Care recommends screening by LDCT for adults aged 55–74 years old who smoke or used to smoke in the last 15 years and smoked 30 pack years. However, there are significant challenges with implementing and adopting such a program, including radiation exposure, poor patient uptake due to lengthy follow-up processes, and resources required to implement and successfully execute a provincial LDCT screening program. Furthermore, LDCT has a very high false-positive rate contributing to further health care resource depletion through unnecessary follow-up procedures and biopsies, increased health care costs, increased patient risk, and patient anxiety. A more straightforward, inexpensive, more accurate early-detection lung cancer test would benefit cancer patients as follows: (1) Earlier, more accurate diagnosis will lead to better patient health for high-risk individuals. Smokers would immediately benefit from low-cost population-based screening for lung cancer. (2) Early detection will save lives and reduce costs related to treatment at advanced stages, targeting the detection of stage I lung cancer (over 53% of patients are asymptomatic), would significantly improve the cure rates as compared to detection at late-stage disease. (3) The dynamics of patient triage and quality of care will change. A metabolomics blood-based test will enable physicians to make evidence-based prevention and treatment decisions. A blood-based test makes sample collection very simple, and it is more likely to encourage patient participation in screening programs. (4) Patient anxiety will be reduced. Obtaining the result of a lung cancer test within hours will decrease the anxiety associated with waiting. If the test is positive, patients will be placed in a correct health care stream for immediate treatment. This test would be expected to fit into the physician’s workflow and current programs quickly.

There are bottlenecks associated with the identification and application of metabolomics. Outstanding concerns preventing the widespread clinical use of metabolomics as a diagnostic/predictive tool are the scalability of data interpretation, the standardization of sample handling practice, and e-infrastructure [120]. However, as these issues become resolved, it is conceivable that the routine utilization of metabolomics at the patient and population levels will constitute an integral part of future healthcare provision [120] and the improved management of patients with cancer [121].

## 7. Conclusions

Cancer is a metabolic disease caused by mutations in key metabolic pathways and key metabolic regulators. Metabolomics has led to identifying several oncometabolites and many high-performing cancer metabolite biomarkers. Thus, the use of metabolomic assays and multi-metabolite markers allows for the rapid, accurate diagnosis and monitoring of not only lung cancer but has the potential applicability in multiple types of cancer. The utility of metabolomics as a fast, accurate, cheap liquid biopsy technique in cancer is under-exploited and thus represents an enormous opportunity in cancer diagnostics and prognostics. We hope the nature of this review contributes to existing knowledge in the field of metabolomics in cancer and how it can be utilized as a robust diagnostic tool for the early detection of lung cancer. Therefore, developing a low-cost, high-throughput metabolomic test will make early-stage lung cancer screening feasible and affordable globally, where survival rates are low, particularly for at-risk populations. In addition, such a paradigm shift in lung cancer screening will make blood-based testing routines accessible to those patients at the highest risk for lung cancer and serve as a viable alternative to molecular and proteomic-based markers for the early detection of lung cancer.

## Figures and Tables

**Table 1:** TNM of malignant tumor classification.

Tumor Size (cm)		Lymph Nodes	Metastasis
T1: <3		N0: no lymph nodes	M0: no
T2	T2a: 3–5 T2b: 5–7	N1: ipsilateral bronchopulmonar/hilar lymph nodes	M1: present
T3: >7		N2: ipsilateral mediastinal/subcarinal lymph nodes	
T4 Invasion Mediastinal organs Vertebral bodies		N3: contralateral/hilar/mediastinal supraclavicular lymph nodes	
**Stage I**		**Stage II**	
Ia: T1, N0, M0		IIa: T2b, N0, M0	
Ib: T2a, N0, M0		IIa: T1, N1, M0	
		IIa: T2a, N1, M0	
		IIb: T2b, N1, M0	
		IIb: T3, N0, M0	

(Adapted from Nooreldeen and Bach [25]). For primary tumor, T0 = No tumor has been found or the original tumor cannot be detected; T1 to T4= Indication of increasing tumor size and extent, i.e., T1, a small tumor, T3 a larger tumor. For lymph nodes, N0 = no tumors in the lymph nodes; N1 to N3 stand for location and number of affected regional lymph nodes. Tumors found in lymph nodes that are not in the drainage area of the affected organ are regarded as distant metastases; for metastases, M0 = no distant metastases observed, M1 = distant metastases found. Stage I refers to presence of a small cancer and only in one area. This is also called early-stage cancer. Stage II means the cancer is larger and has grown to nearby tissues or lymph nodes. Addition of the lowercase letter a or b (i.e., T2a, T2b) in the classification has been used to subdivide the tumour, lymph nodes or metastasis categories to make them more specific.

**Table 2:** Dysregulated metabolites among various types of lung cancer.

Sample	Type of Cancer										Relevant Metabolites	Reference
	Ad	LCC	N	Sq	S	LC	M	H	P	Com		
Tissue												
	14							20 *	A	DS	Early stage (selected metabolites that differentiate the groups): creatine, creatinine, GP-N-heptacenyl EA, GP-N-heptadecanoyl EA, GP-NAE, GP-N-hexodecenyl EA, GP-N-octadecanoyl EA, GP-N-octadecenyl EA, DHTH, LysoPE P-16:0, and xanthine.	[80]
	33				54	12		20 *	A	DS	Advanced stage (selected metabolites that differentiate the groups): arachidonic acid, LysoPC 16:0 plasm, oleic acid, PC 18:1 plasm/0:0, PC 32:0 plasm, adrenic acid, DPA, LysoPC 16:2 plasm, PC 34:2(OH), PE 0–18:2/0:0, and P-18:1/0:0.	
Plasma												
	21				18			20 *	A	DS	Early stage (selected metabolites that differentiate the groups): PC 15:0/22:6 and PC 18:1/22:6.	[80]
	11	7			15						Advanced stage (selected metabolites that differentiate the groups): deoxycholic acid, glycocholic acid, PE P-18:1/20:5, PE P-16:0/22:5, PC O-16:0/18:2, PE P-19:1, PC 40:1, PC 38:0, linoleic acid, and arachidonic acid.	
	6		6	7	5		2	29	B	DC	Valine, LysoPC (18:2), decadienyl-L-carnitine (C10:2) phosphatidylcholine, acyl-alkyl C36:0 (PC aa C36:0), phosphatidylcholine diacyl C32:2 (PC aa C32:2, phosphatidylcholine diacyl C30:2 (PC aa C30:2), spermine, putrescine, and diacetylspermine.	[81]
	31							28	C	DC	Alanine, glutamine, glycine, threonine, 5-hydroxytryptophan, 5-methoxytryptophan, L-arginine, proline, *N*(6)-methyllysine, deoxycholic acid glycine conjugate, PC (34:4), PE (34:2), PE (36:1), PE (36:2), PE (36:4), PE (38:4), PE (38:6), PE (40:4), PE (40:5), PS (38:4), ceramides (42:0), palmitic acid, linoleic acid, oleic acid, amylose, maltitol, testosterone sulfate, androsterone sulfate, pregnenolone sulfate, 3-hydroxybutyric acid, pipecolic acid, uric acid, bilirubin, ubiquinone, ubiquinol, 3,4,5-trimethoxycinnamic acid, and *N*-palmitoleoyl ethanolamide.	[82]
	12			9	4			25	D	DC	LDL/VLDL, β-hydroxybutyrate, unsaturated lipids, acetoacetate, α-glucose, β-glucose, lactate, glutamate, glutamine, tyrosine, histidine, choline, phosphocholine, glycerophosphocholine, betaine, and TMAO.	[83]
									E		Aspartate, asparagine, glutamate, glutamine, cysteine, methionine, isoleucine, leucine, and tryptophan.	
Biofluid plasma												
	110			46				60	B	DC	β-hydroxybutyric acid, LysoPC (20:3), PC ae (C40:6), citric acid, fumaric acid, and carnitine.	[84]
BALF												
	8		6	7	3			30 ^	F	DC	Lactic acid, acetic acid, glycerol, L-glycine, L-aspartate, L-proline, L-glutamine, fructose, phosphoric acid, isocitric acid, inositol, galactose, palmitic acid, stearic acid, inosine, and oleic acid.	[85]
Serum												
	9			9	6	8		29	F	DC	L-valine, L-glycine, tartaric acid, L-serine, L-threonine, uridine, malonic acid, L-proline, L-cysteine, L-glutamine, L-phenylalanine, fructose, phosphoric acid, isocitric acid, L-asparagine, inositol, L-ornithine, deoxy-glucose, glucose, palmitic acid, uric acid, stearic acid, L-cystine, myristic acid, margaric acid, and arachidonic acid.	[85]
	15	3		12				30	G	DC	8-OH guanine, phenylglyoxylic acid, L-pipecolic acid, L-carnitine, caffeic acid 3-sulfate, dimethyl fumarate, L-adrenaline, adrenosterone, 3-OH-3-methyl-glutaric acid, acetylcarnitine, L-valine, uric acid, oxalosuccinic acid, cortisol, Prasterone sulfate, uridine, sphinganine, sphingosine, phosphorylcholine, PC(15:0), glycerophospho-*N*-arachidonoyl ethanolamine, palmitoyl -L-carnitine, PC(16:0), oleamide, glycerophospho-*N*-oleyl ethanolamine, PC(17:0), 1,25-hydroxyvitamin D3, arachidyl carnitine, PC(18:0), glycocholic acid, γ-linolenic acid, and linoleic acid.	[86]
	15	3		12				30	H	DC	Lactic acid, alanine, α-hydroxyisobutyric acid, α-hydroxybutyric acid, L-valine, urea, serine, L-leucine, phosphate, L-isoleucine, glycine, L-threonine, aminomalonic acid, pyroglutamic acid, 2,3,4-trihydroxybutyric acid, L-phenylalanine, tetradecanoic acid, glucose, palmitic acid, myo-inositol, 9,12-octadecadienoic acid, oleic acid, and cholesterol.	
	43							43	H	DC	Maltose, maltotriose, cystine, 3-phosphoglycerate, citrulline, pyrophosphate, tryptophan, adenosine-5-phosphate, Bin_226841, Bin_367991, Bin_715929, Bin_299216, and cellobiotol.	[87]
			65					65	I	DC	Cysteine, serine, glycine, leucine, aspartic acid, cholesterol, 2-hydroxyglutaric acid, and 1- monooleoylglycerol.	[88]
	4		3	5	5			30	J	DC	2015 dataset: valine, leucine, ornithine, methionine, histidine, phenylalanine, arginine, citrulline, tyrosine, aspartate + asparagine, C3-carnitine, C4-carnitine, C5-carnitine, C8-carnitine, C14-carnitine, and C16-carnitine.2017 dataset: glycine, valine, leucine, methionine, histidine, citrulline, and arginine.	[89]
						35		70	K	DC	Bisphenol A, retinol, L-proline.	[90]
	25			18				50	L	DC	Hypoxanthine, inosine, L-tryptophan, indoleacrylic acid, acyl-carnitine C10:1, and LysoPC (18:2).	[91]
						31		29	H	DC	Indole-3-lactate, erythritol, adenosine-5-phosphate, paracetamol, threitol, oxalic acid, fructose, inosine, naproxen, lyxose, caprylic acid, dodecanol, and cystine.	[92]
						50		41	H	DC	Cholesterol, oleic acid, myo-inositol, 2-hydroxybutyric acid, 4-hydroxybutyric acid, and pelargonic acid.	[93]
Sputum												
						23		33	C	DC	Isobutyl decanoate, putrescine, diethyl glutarate, cysteamine, hexanal, cysteic acid, and hydropyruvic acid.	[94]
Urine												
	9			9	6	8		29	F	DC	L-alanine, acetic acid, malonic acid, urea, L-glycine, succinic acid, glyceric acid, L-serine, L-threonine, butanoic acid, threonic acid, creatinine, glutaconic acid, L-aspartate, ribonic acid, adipic acid, arabitol, aconitic acid, phosphoric acid, isocitric acid, hippuric acid, purine, inositol, gluconic acid, sorbitol, glucaric acid, galactaric acid, palmitic acid, uric acid, and stearic acid.	[85]
	51		10		14			78	M	DC	Creatine riboside and *N*-acetylneuraminic acid.	[95]
	13			22		3		42	N	DC	Glycyl-glycine, 7-ethyl-5,6-dihydro-1,4-dimethylazulene, 4-pyridoxic acid, crithmumdiol, 4-(methylnitrosamino)-1-(3-pyridyl)-1-butanone, 1-methylhistidine, indoxyl sulfate, tryptophan, deacetyldiltiazem, maltulose, porson, 8-hydroxynevirapine, austinol, dehydroepiandrosterone sulfate, dehydroepiandrosterone 3-glucuronide, 5-alpha-dihydrotestosterone glucuronide, dulciol C, imidazoelactic acid, glutamine, lyciumoside IV, neuromedin C 1–8, withaphysacarpin, noradrenochrome o-semiquinone, S-prenyl-L-cysteine, tetrahydroaldosterone-3-glucuronide, ax-4′-hydroxy-3′-methoxymasin, dulxanthone, androsterone sulfate, cortolone-3-glucuronide, tyrosine, xanthosine. tyrosyl tyrosine, 3 beta-dihydroxymarasmene, indoxyl, and *cis*-zeatin-O-glucoside.	[96]

Ad, adenocarcinoma; LCC, large cell carcinoma; N, non-small-cell lung carcinoma; Sq, squamous cell carcinoma; LC, undefined lung cancer type; S, small-cell carcinoma; M, metastasis; H, healthy controls; P, platform used; Com, comparison; DS, comparison between different disease subgroups; DC, comparison between the disease and the control; BALF, bronchoalveolar lavage fluid; *, COPD patients; ^, non-cancerous lung disease; A, LC-QTOF-MS; B, ABI4000 Qtrap tandem MS; C, Agilent 6890 GC-MS + Agilent 6540 quadrupole-time of flight (Q-TOF); D, ^1^H NMR; E, Agilent 1260 Rapid Resolution LC; F, Trace GC Ultra GC-MS; G, Agilent 6530 accurate-Mass Q-TOF/MS; H, Agilent 7890A GC-MS; I, QP2010 GC−MS; J, Agilent 6430 triple quadrupole-MS; K, Agilent Q-TOF 6550; L, SCIEX X500R QTOF-MS; M, Waters quadrupole time-of-flight (QTOF)-MS; N, Waters SNAPT G2-Si Q-TOF/HRMS. The metabolites described in the Relevant Metabolites column include both discovery and validation datasets.

## Data Availability

Not appliable.
